# A Unifying Pathophysiological Account for Post-stroke Spasticity and Disordered Motor Control

**DOI:** 10.3389/fneur.2019.00468

**Published:** 2019-05-10

**Authors:** Sheng Li, Yen-Ting Chen, Gerard E. Francisco, Ping Zhou, William Zev Rymer

**Affiliations:** ^1^Department of Physical Medicine and Rehabilitation, McGovern Medical School, University of Texas Health Science Center – Houston and TIRR Memorial Hermann Hospital, Houston, TX, United States; ^2^Shirley Ryan Ability Lab, Chicago, IL, United States

**Keywords:** spasticity, motor control, synergy, stroke, reticulospinal tract

## Abstract

Cortical and subcortical plastic reorganization occurs in the course of motor recovery after stroke. It is largely accepted that plasticity of ipsilesional motor cortex primarily contributes to recovery of motor function, while the contributions of contralesional motor cortex are not completely understood. As a result of damages to motor cortex and its descending pathways and subsequent unmasking of inhibition, there is evidence of upregulation of reticulospinal tract (RST) excitability in the contralesional side. Both animal studies and human studies with stroke survivors suggest and support the role of RST hyperexcitability in post-stroke spasticity. Findings from animal studies demonstrate the compensatory role of RST hyperexcitability in recovery of motor function. In contrast, RST hyperexcitability appears to be related more to abnormal motor synergy and disordered motor control in stroke survivors. It does not contribute to recovery of normal motor function. Recent animal studies highlight laterality dominance of corticoreticular projections. In particular, there exists upregulation of ipsilateral corticoreticular projections from contralesional premotor cortex (PM) and supplementary motor area (SMA) to medial reticular nuclei. We revisit and revise the previous theoretical framework and propose a unifying account. This account highlights the importance of ipsilateral PM/SMA-cortico-reticulospinal tract hyperexcitability from the contralesional motor cortex as a result of disinhibition after stroke. This account provides a pathophysiological basis for post-stroke spasticity and related movement impairments, such as abnormal motor synergy and disordered motor control. However, further research is needed to examine this pathway in stroke survivors to better understand its potential roles, especially in muscle strength and motor recovery. This account could provide a pathophysiological target for developing neuromodulatory interventions to manage spasticity and thus possibly to facilitate motor recovery.

## Introduction

Stroke is a leading cause of adult disability (1). According to the centers for disease control and prevention (CDC), ~800,000 people have a stroke every year in the United States, resulting in a total of 7 million stroke survivors. Motor impairments are common, seen in about 80% of stroke survivors. Motor impairments mainly include weakness, spasticity, abnormal motor synergy, and disordered motor control. Spasticity and its related abnormal joint postures often interact with weakness and loss of dexterity, resulting in disordered motor control and functional limitations, such as inability to grasp, reach, walk, and transfer. Collectively, these motor impairments result in difficulties in mobility and activities of daily living, and limit their vocational and social participation in more than half of stroke survivors at age 65 and over (1). As such, these motor impairments not only have downstream effects on stroke survivors' quality of life, also lay substantial burdens on the caregivers and society (2). Numerous pharmacological agents and physical modalities and interventions have been utilized for stroke motor rehabilitation programs, but with varying degrees of success. Motor recovery after stroke still remains a clinical challenge. One of the biggest challenges is that the mechanisms underlying motor recovery are not well understood.

Neural plasticity plays an important role in motor recovery as well as development of motor complications, such as spasticity after stroke (3). Post-stroke plastic changes occur in ipsilesional, contralesional motor cortices, and subcortical areas, such as primarily pontomedullary reticular formation (PMRF) (4–21). It is largely accepted that plasticity in the ipsilesional primary motor cortex (iM1) primarily contributes to recovery of motor function, while contributions of contralesional primary motor cortex (cM1) are not completely understood. The role of cM1 reorganization depends on lesion location, size and motor impairment (20, 22). It is likely mediated by ipsilateral cortico-reticulospinal (RS) projections and uncrossed ipsilateral CST from contralesional motor cortex (19, 22–24). As a consequence of damages to iM1 and its descending pathways, both animal studies and human imaging studies suggest that there is increased excitability in the brainstem reticular system and its descending reticulospinal tract (RST) (25–27). Both animal and human studies support the maladaptive role of RS hyperexcitability in spasticity. However, animal studies have demonstrated the important role of RST in motor recovery, while its role remains controversial in human stroke studies. In this article, we first summarize experimental evidence supporting upregulations of RS excitability. The potential roles of RST in post-stroke recovery and spasticity are then compared in both animal and human stroke studies. A unifying account is proposed to better understand the brainstem roles and to consolidate controversial findings for spasticity and disordered motor control.

## Upregulation of Reticulospinal Excitability After Stroke

The reticulospinal (RS) system is another major descending system, in addition to CST. The RS system is best known for its role in posture and locomotion (28), but it also recruits both proximal and distal muscles of the upper extremity bilaterally (29), including the finger muscles (30, 31). There are two descending RS tracts with distinctly different origins. The dorsal RST originates from the dorsolateral reticular formation in the medulla, and receives facilitation from the motor cortex via corticoreticular fibers. The lateral CST and cortico-reticulo-spinal tract descend adjacent to each other in the dorsolateral funiculus at the spinal level. The medial RST originates primarily from the pontine tegmentum with connections to PMRF. The medial RST descends along with the vestibulospinal tract (VST) in the ventromedial cord. The dorsal RST provides dominant inhibitory effects to spinal reflex circuits, while medial RST and VST provide excitatory inputs. Therefore, medial and lateral RSTs provide balanced excitatory and inhibitory inputs to spinal motor neuron network. In the context of stroke with cortical and internal capsular lesions, damages often occur to both CST and corticoreticular tracts due to their anatomical proximity. This leaves the facilitatory medial RST and VST unopposed, thus hyperexcitability [see reviews in (25–27, 32)]. However, due to technical difficulties, activities of brainstem nuclei and RST excitability cannot be localized and assessed directly in stroke survivors, even with most advanced technologies (33–37).

The RST hyperexcitability in humans has been assessed indirectly through acoustic startle reflex (ASR). ASR is an involuntary motoric response to unexpected loud auditory stimuli (38). The proposed circuit of the ASR in humans involves the cochlear nucleus, the caudal pontine reticular nuclei, the motoneurons of the brainstem, and the spinal cord activated through the medial RST (39–41). ASR has been established in the literature to investigate RST excitability in healthy and stroke subjects (31, 42–52). In stroke survivors with cerebral infarcts, normal ASR motoric responses could be elicited in flaccid muscles in the acute phase, however no response from the same muscles to magnetic cortical stimulation of the primary motor cortex was elicited in these patients (42), suggesting that the circuit of ASR remained intact in these patients and not under cortical control of iM1 and its descending pathways. In a different study in chronic stroke, exaggerated ASR responses were observed in spastic muscles (43), suggesting RST hyperexcitability. In a recent study (52), we compared ASR responses in chronic stroke at different stages of motor recovery (Flaccid, Spastic, and Recovered; Flaccid = those who remain flaccid; Recovered = those who have a history of spasticity but have recovered and have isolated voluntary movement). We found that ASR responses were within normal limits in stroke survivors without spasticity (Flaccid or Recovered). However, exaggerated ASR responses were frequently observed in spastic subjects bilaterally, but more evidently (earlier and longer duration) on the impaired side than on the non-impaired side. These results suggest that RST hyperexcitability occurs in the Spastic stages, but not in the Flaccid or Recovered stages in chronic stroke.

## Controversial Roles of Reticulospinal Tracts in Motor Recovery and Disordered Motor Control

Accumulated evidence from animal studies appears to support the role of RS hyperexcitability in motor recovery after CST damage due to stroke (29, 53–56). Riddle and Baker (56) reported that medial RS and corticospinal pathways descended in parallel and had largely overlapping effects on spinal interneurons and motoneurons in non-human primates; importantly, responses from spinal motoneurons to stimulation of either pathway at supraspinal levels were of similar amplitudes during a reach and grasp task. Buford and colleagues further reported a significantly increased RS motor output that contributed to recovery of voluntary motor control in monkeys with significantly damaged primary motor cortex and its descending CST (57, 58). For example, Buford et al. reported that, reaching was severely impaired after a substantial focal ischemic M1 lesion in an adult macaque. However, reaching performance had a near normal recovery after 12 weeks of intensive therapy. This improvement was paralleled with significantly increased output from the reticulospinal system, while little to no change was observed in both ipsilesional and contralesional M1 (58). Therefore, strengthening the existing intact RS projections is a plausible mechanism for motor recovery as seen in these animal models (56–60).

These findings do not translate into clinical practice. Studies with stroke survivors have demonstrated that RST may not always be beneficial (22, 61). Byblow et al. suggested that the contralesional motor cortex facilitates the descending ipsilateral cortico-reticulo-spinal projections or cortico-reticulo-propriospinal projections after stroke. These projections may contribute to motor recovery in patients with severe paresis, but not in the less impaired limb (22, 62).

The possible contributions to force production from cM1 and its descending ipsilateral cortico-reticulo-spinal pathways seem insignificant, however. The contributions were examined in a recent TMS study (63). TMS to cM1 was delivered during isometric elbow flexion at submaximal levels on the spastic-paretic side in chronic stroke and in healthy subjects. The TMS-induced force increment was significant greater only at 10% of maximal voluntary contraction tasks in stroke subjects than in healthy controls. No significant difference in the force increment was found at higher force levels. In a recent study, during isometric elbow flexion tasks, the force increment induced by stimulation of RST via startling acoustic sound in stroke survivors with spastic elbow flexors was not significantly different from the increment in neurologically intact subjects (64). Taken together, these findings indicate that RST hyperexcitability does not provide additional contributions to voluntary elbow flexion force production in chronic stroke survivors.

On the other hand, RS hyperexcitability is associated with abnormal motor synergy and disordered motor control in chronic stroke survivors. In a DTI study, RST reorganization and strengthening is significantly correlated with impairments and abnormal synergy, not motor recovery (24). In a series of studies by Dewald and colleagues (24, 65–71), they have consistently reported involvement of RS hyperexcitability in abnormal synergy in shoulder, elbow, wrist and finger movement on the paretic side in chronic stroke with moderate to severe motor impairment. Specifically, they provide evidence that contralesional cortico-reticulospinal pathways are progressively recruited, but they do not contribute to discrete voluntary movement (70).

RS hyperexcitability seems to be maladaptive in the course of complete motor recovery. In a recent longitudinal study in 2018 (21), the authors tracked the time course of mirror movement in the non-paretic hand during individual finger movement of the paretic hand in stroke survivors since 2 weeks post stroke. They reported mirroring in the non-paretic hand was exaggerated early after stroke, but progressively improved over the year. The improvement paralleled individuation deficits in the paretic hand in the time course. However, these changes were not concomitantly accompanied by any evidence of cortical mechanisms according to fMRI data. The authors attributed these changes to upregulation of subcortical mechanisms, particularly RS hyperexcitability in the early recovery phase. During the course of recovery, improvement in mirroring reflects the reliance on the capacity of cortical sensorimotor areas in both hemispheres to re-gain modulatory influences on the RST.

## The Role of RST Hyperexcitability in Post-Stroke Spasticity

Post-stroke spasticity is a common phenomenon of velocity-dependent increase in resistance when a joint is passively stretched. It is accepted that spasticity is mediated by exaggerated spinal stretch reflex (25–27, 32, 72, 73). Animal lesion studies in last century have provided strong experimental evidence to support the role of RST hyperexcitability in spasticity. For example, isolated lesions to CST only produce weakness, loss of dexterity, hypotonia, and hyporeflexia, instead of spasticity (74–76). Surgical section of unilateral or bilateral VST in the anterior cord has little effect (77) or a transient effect (78) on spasticity. With more extensive cordotomies that damage the medial RST, spasticity is dramatically reduced (78). In another study, Burke et al. provided evidence that spasticity and decerebrate rigidity are differentially mediated through RST and VST projections (79).

Overall, findings from studies with human subjects are consistent with findings from animal studies on the role of RST for spasticity. As mentioned earlier, there are exaggerated acoustic startle reflexes in stroke survivors with spasticity (43, 52). The RST plays an important role in maintaining joint position and posture against gravity (28). The findings of high correlations between the resting joint of elbow joint and severity of spasticity (clinical and biomechanical measurements) (80) suggest that post-stroke spasticity is strongly related to RS hyperexcitability and its antigravity effects.

The descending medial RST inputs to the spinal motor neurons from medial PMRF are primarily mediated by the monoamines serotonin (5-HT) and norepinephrine (NE). The monoaminergic inputs via unopposed hyperexcitable RST provide powerful neuromodulatory changes of spinal motor neurons, greatly increasing their excitability and facilitating persistent inward currents (PIC) (81–83). The PIC is a depolarizing current generated by voltage-activated channels that tend to remain activated, thus associated with a plateau behavior (84). PICs are associated with subthreshold depolarization of spinal motor neurons, and hyperactive stretch reflexes in the spastic-paretic limb following stroke, thus mediating spasticity. A serotonergic agent (estitalopram) can augment spasticity (85), while an anti-serotonergic agent (cyproheptadine) facilitates relaxation time of spastic muscles (86). Reduction in descending NE drive via administration of tizanidine has shown to improve independent joint control in chronic stroke survivors with moderate to severe motor impairments (87).

Given unilateral nature of VST projections (88), the role of VST in spasticity was recently tested in chronic stroke (89, 90), in which VST was stimulated via high-level acoustic stimuli (130 dB). The results showed a strong correlation between triggered responses and overall severity of spasticity, thus suggesting the role of hyperexcitability of VST in spasticity. Yet this level of acoustic stimuli is also likely to activate RST pathways (39, 91). The role of VST in spasticity cannot be ruled out in human subjects. It is possible that VST affects spasticity via the VST-RST connectivity as mentioned above (92). As mentioned earlier, the findings from animal study do not support the role of VST in spasticity. Findings from advanced neuroimaging study in chronic stroke with severe motor impaired fail to reveal increased VST size as well (24).

## A Unifying Account for Spasticity, Motor Recovery, and Disordered Motor Control

In summary, findings from both animal studies and studies with human subjects support the role of RST hyperexcitability in post-stroke spasticity. In contrast, the compensatory role of RST hyperexcitability in motor recovery is only documented in animal studies, while RST hyperexcitability is more likely related to abnormal synergy and disordered motor control, but not to recovery of voluntary movement in stroke survivors. Both RST and CST work together to recruit muscles during voluntary movement. RST is known of particular importance in concert with actions of the ipsilateral CST (93). For example, in chronic stroke survivors, it was found that the fiber volume of ipsilateral corticoreticular projections from the contralesional hemisphere was increased, and such change was correlated to walking ability (19). In another study (94), findings suggested a relationship between increased activity in the contralesinal cortical areas (M1, premotor, and primary sensory cortex) and spasticity mitigation in response to motor learning therapy in chronic stroke. However, efforts and strategies to promote motor recovery have focused mainly on iM1 and cM1, for example, they are targets of non-invasive brain stimulation (95–97). The RST involvement is considered beneficial in those with severe motor impairment (22). In contrast, RST hyperexcitability has been emphasized to likely mediate post-stroke spasticity (25–27, 32). It lacks a theoretical framework to understand the role of the brainstem reticulospinal system and its interactions with the corticospinal motor system in motor recovery, disordered motor control, and spasticity.

**Figure 1:**
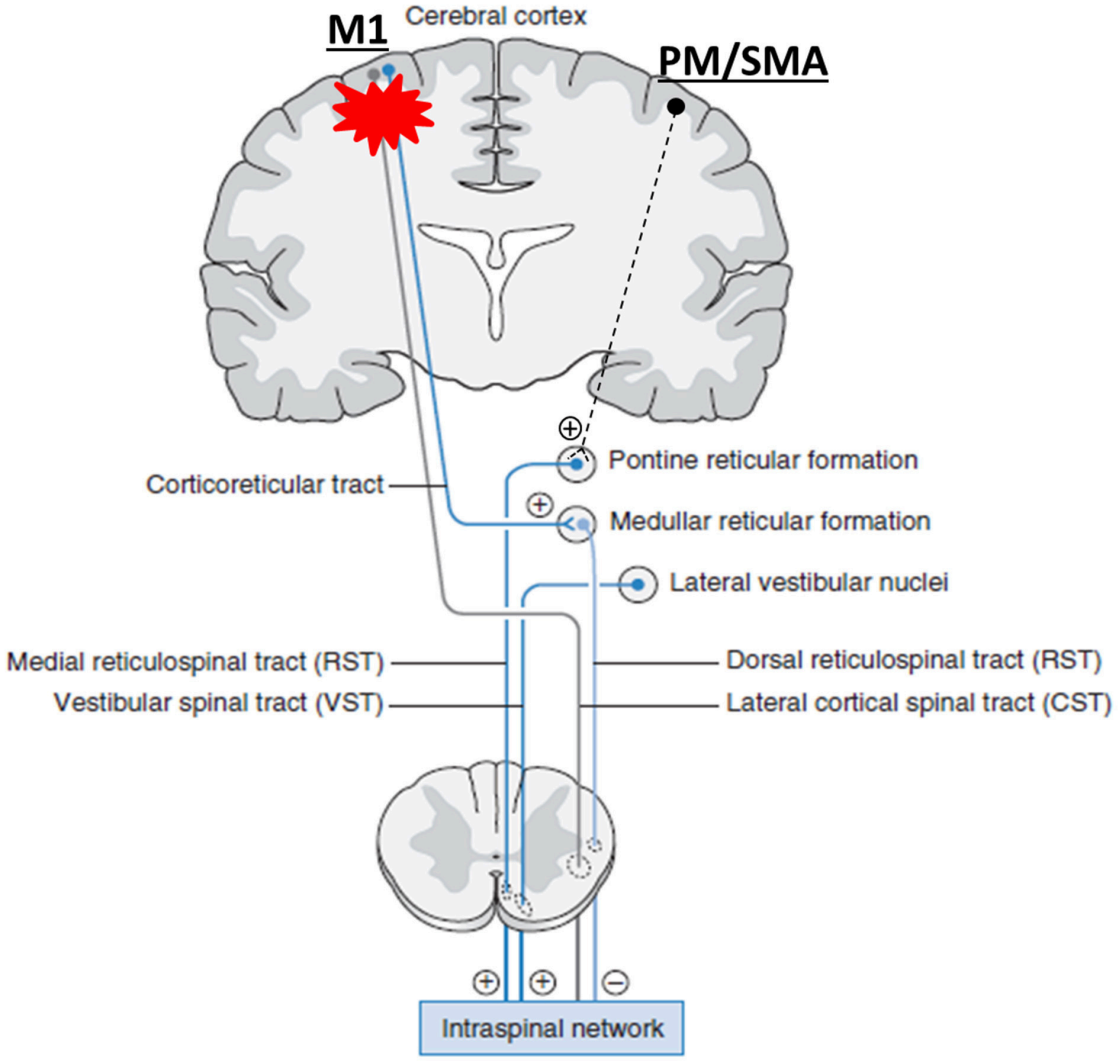
A unifying pathophysiological account for post-stroke spasticity, abnormal synergy, and disordered motor control. For the medial PMRF, it receives inputs primarily from ipsilateral premotor (PM) and supplementary motor area (SMA), and descends ipsilaterally. This medial cortico-reticulo-spinal tract (CRST) provides excitatory descending inputs to spinal motor neurons. The dorsolateral PMRF receives inputs primarily from contralateral primary motor cortex (M1). This dorsal CRST provides inhibitory descending inputs to the spinal motor circuitry. When damages occur to the corticospial tract (CST) and CRST after stroke on one hemisphere (red asterisk), their output signals diminish. Subsequently, the medial CRST excitability of the contralesional hemisphere becomes unopposed, upregulated gradually, and hyperexcitable. Eventually, spinal motor neurons are hyperexcitable, or may be spontaneously firing. (+): excitatory; (–): inhibitory. Note: other descending pathways are not illustrated. They are considered either insignificant or connected with the reticulospinal tract [modified from Francisco and Li (98)].

Recent research findings provide new insights into understanding the role of RST and its interactions with CST in stroke survivors. It was believed that the ventromedial reticular formation in the medulla receives the excitatory inputs vial corticoreticular projections from the contralateral M1 and gives out dorsal RST and descends ipsilaterally next to the lateral CST; while the medial RST originates diffusely within medial pontomedullary reticular formation (PMRF) (25–27, 32). After stroke-related damage to the M1 and its descending CST and corticoreticular projections, the medial RST becomes unopposed and gradually hyperexcitable, providing excitatory inputs to the spinal motor neurons (see Figure 1 without dashed projection from PM/SMA) (25–27, 32). Recent studies demonstrate that cortico-reticulo-spinal projections are bilateral, but have laterality dominance (99–101). For the medial PMRF, it receives inputs primarily from ipsilateral premotor (PM) and supplementary motor area (SMA), and descends ipsilaterally to the spinal cord. This medial cortico-reticulo-spinal tract (CRST) provides excitatory descending inputs to spinal motor neurons. The dorsolateral PMRF receives inputs primarily from contralateral primary motor cortex (M1). This dorsal CRST provides inhibitory descending inputs to the spinal motor circuitry. Following focal cortical lesions in monkeys, there are reports of upregulation of contralateral SMA/PM-corticoreticular projections (100–102). Taken these findings into consideration, we propose a unifying account in understanding the role of RST hyperexcitability in post-stroke spasticity, abnormal synergy, and disordered motor control. Figure 1 schematically illustrates this account. As compared to previous accounts, the novel addition is that the medial PMRF receives excitatory inputs primarily from the ipsilateral PM and SMA of the contralesional cortex. In addition to further support the role of RST hyperexcitability in spasticity as in previous accounts, this unifying account provides a theoretical framework to understand the role of RST hyperexcitability and its interactions with bilateral motor cortices in motor recovery and abnormal synergies.

### Abnormal Motor Synergy and Disordered Motor Control

The RST has diffuse and divergent projections to multiple synergic muscles. Given the resultant hyperexcitable spinal motor neurons, attempts to voluntarily activate a spastic muscle often result in synergistic activation of multiple spastic muscles, i.e., abnormal motor synergy. This phenomenon has been consistently reported by Dewald and colleagues (24, 65–71). The abnormal coupling of muscle activation has also been found between upper and lower extremities (103) and between two upper extremities (104). Therefore, motor synergies are simplified and more stereotyped gait patterns are seen in stroke survivors with spasticity (105). Similarly, inter-joint coordination between spastic muscles is impaired, leading to disordered motor control (106–109). Furthermore, it is conceivable that PM/SMA and cM1 are both activated during voluntary elbow flexion on the less-affected side, thus subsequently activating ipsilateral spastic muscles involuntarily via the PM/SMA-CRST projections, i.e., motor overflow (110).

### Motor Recovery

RST excitability increases along with the emergence of post-stroke spasticity and subsides in the recovered stage. In addition, no exaggerated motor overflow is observed in stroke survivors with recovered motor function (52). This may be related to the fact that ipsilesional M1 evolves and regains cortical control of motor function as motor recovery progresses (13). As such ipsilesional M1 regains its neuromodulatory inputs to PMRF and rebalances RST influences to spinal motor networks. This could also account for disappearance of spasticity in the recovered, late Brunnstrom stages (111). However, this seemingly maladaptive RST hyperexcitability is conceivable to facilitate movement via the ipsilateral CRST. In a recent study on chronic stroke survivors with severe motor impairment, facilitatory stimulation via repetitive TMS to the dorsal premotor area on the contralesional hemisphere improved reaching time on the paretic side (20). It remains unclear whether this improvement relates to a stronger synergistic activation or improved isolate movement and strength.

### Muscle Strength

There is evidence that startling acoustic stimulation enhances maximum force production in healthy subjects via stimulation of RST (112, 113). In chronic stroke survivors, startling acoustic sound stimulation does not cause additional force increment as compared to healthy subjects (64). Given different cortical origins for medial and dorsal PMRF areas (Figure 1), it is likely that the RST projections function to tune and modulate motor commands (114), rather to compensate for them. The StartReact phenomenon could support this view. The StartReact phenomenon refers to an early release of a motor plan during simple reaction time tasks in the presence of startling acoustic stimulation (115). This phenomenon is seen in stroke survivors as well (46, 48). However, no early release of motor plan is observed when no motor plan is ready in a choice reaction time task (116), or a Go/No-go reaction time task (117).

### Concluding Remarks

Based on recent advances in animal studies and human studies with stroke survivors, we revisit and revise the previous theoretical framework and propose a unifying account. This account highlights the importance of ipsilateral PM/SMA-cortico-reticulospinal tract hyperexcitability as a result of disinhibition after stroke. This account is able to provide a pathophysiological basis for post-stroke spasticity and related movement impairments, such as abnormal motor synergy, disordered motor control. This account could provide a pathophysiological target for neuromodulatory interventions to manage spasticity, and thus possibly to facilitate motor recovery. Some issues and controversies remain to be further addressed, such as the role of higher cortical centers (e.g., PM and SMA) and bilateral RST projections. The contributions from other descending pathways, especially VST projections need to be further studied. With advances in technologies, further research on these issues is needed to better understand the potential roles of this pathway in stroke motor recovery.

## Author Contributions

SL developed the initial version of the manuscript and created the figure. Y-TC, GF, PZ, and WR critically revised the manuscript and contributed substantially to the manuscript development. All authors read and approved the final manuscript.

### Conflict of Interest Statement

The authors declare that the research was conducted in the absence of any commercial or financial relationships that could be construed as a potential conflict of interest.
